# Demographic predictors of hepatitis C virus viremia among seropositive individuals in Ethiopia: retrospective analysis and implications for testing algorithms

**DOI:** 10.11604/pamj.2026.53.147.50389

**Published:** 2026-04-01

**Authors:** Mesfin Nigussie, Kifle Tilahun

**Affiliations:** 1 International Clinical Laboratories, Addis Ababa, Ethiopia

**Keywords:** Hepatitis C virus, RNA detectability, diagnostic algorithms, age factors, sex factors

## Abstract

**Introduction:**

hepatitis C virus (HCV) screening efficiency in resource-limited settings is compromised by variable ribonucleic acid (RNA) detectability among seropositive individuals. Understanding the demographic predictors of RNA positivity is crucial for optimizing testing algorithms in high-burden populations such as Ethiopia.

**Methods:**

we conducted a retrospective analysis of 792 anti-HCV seropositive treatment-naive individuals in Ethiopia. Multivariate logistic regression with comprehensive diagnostics was used to assess the independent associations of age and sex with HCV RNA status. Assay specificity was enhanced through duplicate testing and elevated signal-to-cutoff thresholds (S/CO ≥5.0).

**Results:**

the overall RNA positivity rate was 69.7% (552/792). Multivariate analysis revealed that each additional year of age increased the odds of RNA positivity by 3.1% (aOR = 1.031, 95% CI: 1.019-1.042, P <0.001), whereas female sex was independently associated with 44.6% greater odds (aOR = 1.446, 95% CI: 1.060-1.973, P = 0.020). Subgroup analysis revealed that young males (<30 years) had lower RNA positivity (37.8%, 95% CI: 24.1-53.9%) than young females did (50.0%, 95% CI: 36.6-63.4%). RNA positivity was 1.6-fold greater in adults ≥30 years (72.8%) than in younger individuals <30 years (44.8%). Genotype 4 predominated (49.6%) among the RNA-positive cases.

**Conclusion:**

both age and sex independently predict HCV RNA detectability in Ethiopia. The substantially lower RNA positivity among young males suggests that this subgroup may benefit from differentiated testing approaches. Demographic-informed algorithms could optimize resource allocation in HCV elimination programs in low- and middle-income countries (LMICs).

## Introduction

Hepatitis C virus (HCV) infection remains a significant global health burden, with an estimated 58 million people living with chronic infection worldwide [1]. Accurate identification of HCV viremia is essential for treatment initiation and transmission prevention, particularly in the context of global elimination goals [2]. While antibody-based screening remains the primary diagnostic entry point, substantial variability in RNA detectability among seropositive individuals creates diagnostic challenges, especially in resource-limited settings where nucleic acid testing capacity is constrained [3,4]. In sub-Saharan Africa, the prevalence of HCV varies considerably, and diagnostic infrastructure is often limited [5]. Ethiopia, with its predominantly young population [6] and unique HCV genotype distribution (predominantly genotype 4) [7], represents an important setting for optimizing testing strategies. Demographic-informed testing approaches may offer efficiency gains-a consideration often overlooked when strategies are directly adopted from high-income settings [8]. Existing evidence suggests that demographic factors influence HCV RNA detectability, with younger individuals typically showing lower RNA positivity rates, potentially due to immune-mediated clearance mechanisms [9-11]. Additionally, sex-linked biological factors may affect both infection susceptibility and viral persistence [12]. The natural history of HCV includes both chronic persistence and spontaneous clearance, with clearance rates varying by population characteristics [13].

However, population-specific data on demographic predictors of RNA detectability in sub-Saharan African contexts remain limited, particularly from routine clinical settings. This study aims to characterize the demographic patterns of HCV RNA detectability among anti-HCV seropositive individuals in Ethiopia and examine the implications for optimized testing algorithms in resource-constrained elimination programs.

## Methods

**Study design:** this was a retrospective cross-sectional analysis of laboratory data from anti-HCV seropositive individuals.

**Setting:** the study was conducted at International Clinical Laboratories in Addis Ababa, Ethiopia, between January and December 2023. The laboratory serves a network of over 100 healthcare facilities across Ethiopia, representing diverse geographic and demographic populations.

**Participants:** seven hundred and ninety-two (792) treatment-naive adults (age ≥18 years) tested positive for anti-HCV antibodies were referred for confirmatory RNA testing and genotyping. Inclusion criteria were: confirmed anti-HCV seropositivity, no prior HCV treatment, and availability of complete demographic and laboratory data. Exclusion criteria were: incomplete records, inadequate sample volume or quality, or evidence of prior HCV treatment.

**Variables:** the primary outcome variable was HCV RNA detectability (positive/negative). The primary predictor variables were age (analyzed as a continuous variable) and sex (binary: male=0, female=1). Secondary variables included HCV genotype distribution among RNA-positive cases.

**Data sources/measurement:** anti-HCV antibodies were detected using the Abbott Alinity HCV chemiluminescent microparticle immunoassay (CMIA) on the Alinity i system (Abbott Laboratories, Chicago, IL, USA). The assay has a manufacturer-reported sensitivity of 99.7% and specificity of 99.8%. To enhance specificity in our setting, we employed a stringent signal-to-cutoff (S/CO) threshold of ≥5.0 (versus the manufacturer's recommended cutoff of 1.0) and conducted duplicate confirmatory testing for all initially reactive samples. HCV RNA extraction and quantification were performed using the Abbott m2000 system with real-time HCV assay (Abbott Molecular, Des Plaines, IL, USA), with a lower limit of detection of 12 IU/mL and quantification range of 12-100,000,000 IU/mL. The assay is calibrated against the WHO International Standard (NIBSC 96/798) and demonstrates ≥99% sensitivity across major HCV genotypes. Genotyping was performed for all RNA-positive samples using the same platform. Serum samples were stored at -20°C for up to one week prior to HCV RNA testing.

**Bias:** several potential biases were considered. Referral bias may exist as participants were referred from clinical settings, potentially representing a symptomatic or higher-risk population compared to community samples. Misclassification bias was minimized through stringent laboratory methods, including duplicate testing and elevated S/CO thresholds. Information bias was addressed by using standardized laboratory protocols. Confounding by factors such as duration of infection, comorbidities, or behavioral risk factors could not be assessed, as these data were not available in this retrospective design.

**Study size:** all consecutive eligible cases during the study period were included (N=792). No formal sample size calculation was performed, as this was a census of available cases. A post-hoc power analysis indicated that with N=792, the study had 80% power to detect an odds ratio of 1.5 for the sex effect at α=0.05.

**Quantitative variables:** age was analyzed as a continuous variable. For subgroup analyses, age was categorized as <30 years, 30-49 years, and ≥50 years. Sex was analyzed as a binary variable. The Number Needed to Test (NNT) was calculated as 1/(proportion RNA-positive) to assess testing efficiency across subgroups.

**Statistical methods:** descriptive statistics were calculated for demographic and laboratory variables. Bivariate associations were assessed using chi-square tests for categorical variables. Multivariable logistic regression was performed to assess independent associations of age and sex with RNA positivity. An age X sex interaction term was formally tested but excluded from the final model due to non-significance (P=0.411). Model diagnostics included calculation of variance inflation factors (all <5, indicating no multicollinearity concern), the Hosmer-Lemeshow goodness-of-fit test (P=0.42), and receiver operating characteristic (ROC) curve analysis to assess discrimination. Statistical analyses were performed using Python (version 3.12.4) with the statsmodels (version 0.14.1) and pandas (version 2.2.1) packages. Statistical significance was set at P<0.05.

**Ethical consideration:** ethical approval for this study was granted by the Addis Ababa Health Bureau Ethics Review Committee (Protocol A/A/A/10332/227). Informed consent was waived because of the retrospective nature of the study and the use of completely anonymized data, in accordance with national ethical guidelines.

## Results

**Participants:** a total of 792 anti-HCV seropositive individuals were included in the analysis. All participants were treatment-naïve adults referred for confirmatory RNA testing following seropositive antibody results. No participants were excluded due to incomplete data or inadequate samples.

**Descriptive data:** the cohort included 792 individuals with a mean age of 49.0 years (standard deviation ± 14.7; range 18-95 years). The sample comprised 431 females (54.4%) and 361 males (45.6%). Demographic characteristics stratified by RNA status are presented in Table 1.

**Table 1:** demographic characteristics of anti-hepatitis C (HCV) virus seropositive individuals by HCV ribonucleic acid (RNA) status, International Clinical Laboratories, Addis Ababa, Ethiopia, January-December 2023 (N=792)

Variable	RNA-positive (n = 552)	RNA-negative (n = 240)	P value
Female, n (%)	324 (58.7)	107 (44.6)	0.012
Median age (IQR)	50 (41-61)	45 (32-56)	<0.001
Age <30 years, n (%)	91 (16.5)	65 (27.1)	<0.001

RNA: ribonucleic acid

**Outcome data:** overall, 69.7% (n=552) of anti-HCV seropositive individuals had detectable HCV RNA, while 30.3% (n=240) were RNA-negative.

**Main results:** multivariable logistic regression adjusted for both age and sex revealed that both factors were independent predictors of RNA status. The final model demonstrated excellent fit (Akaike information criterion=940.4) and acceptable discrimination (area under the ROC curve=0.631, 95% CI: 0.598-0.665). Each additional year of age increased the odds of RNA positivity by 3.1% (aOR=1.031, 95% CI: 1.019-1.042, P<0.001). Female sex was independently associated with 44.6% greater odds of RNA positivity (aOR=1.446, 95% CI: 1.060-1.973, P=0.020).

Stratified analysis revealed substantial demographic variation in RNA positivity rates (Table 2). Young males (<30 years) presented the lowest positivity rate (37.8%, 95% CI: 24.1-53.9%), while young females presented substantially higher positivity (50.0%, 95% CI: 36.6-63.4%). This sex-based pattern attenuated in older age groups. RNA positivity was 1.6-fold higher in adults aged ≥30 years (72.8%) than in younger individuals (44.8%). This difference translates to a number needed to test (NNT) of 1.4 versus 2.2 to identify one RNA-positive case in older versus younger individuals, respectively.

**Table 2:** hepatitis C virus ribonucleic acid (RNA) positivity rates by age group and sex among anti-HCV seropositive individuals, International Clinical Laboratories, Addis Ababa, Ethiopia, January-December 2023 (N=792)

Age group	Sex	Total (n)	RNA-positive, n (%)	Positivity rate (95% CI)
< 30 years	Female	50	25 (50.0)	50.0% (36.6-63.4)
< 30 years	Male	37	14 (37.8)	37.8% (24.1-53.9)
30-49 years	Female	167	122 (73.1)	73.1% (65.9-79.3)
30-49 years	Male	183	120 (65.6)	65.6% (58.4-72.2)
≥ 50 years	Female	214	177 (82.7)	82.7% (77.1-87.2)
≥ 50 years	Male	141	94 (66.7)	66.7% (58.4-74.1)

**Other analyses:** among the 552 RNA-positive individuals, genotype 4 was predominant (49.6%), followed by genotype 1 (27.2%), genotype 2 (10.9%), genotype 6 (6.9%), genotype 5 (4.0%), and genotype 3 (0.4%). Six samples (1.1%) were classified as indeterminate (RNA positive but untypable). No significant associations were observed between genotype distribution and demographic factors (P>0.05 for all comparisons).

## Discussion

**Key findings:** this analysis of 792 anti-HCV seropositive individuals in Ethiopia reveals distinct demographic patterns in HCV RNA detectability, with important implications for testing algorithms in resource-limited settings. The independent associations of both increasing age and female sex with RNA positivity, coupled with the substantially lower detectability in young males, provide evidence for optimizing testing strategies in elimination programs. The strong, continuous age effect (3.1% increased odds of RNA positivity per year of age) aligns with established literature on age-related declines in immune function [9-11]. Older individuals may have a reduced capacity for spontaneous viral clearance due to immunosenescence, leading to higher rates of chronic infection [14].

**Interpretation:** our finding that female sex remains independently associated with increased RNA positivity after age adjustment contrasts with some previous reports [15] and highlights the importance of population-specific analyses. This association may reflect sex-based immunological differences or hormonal influences on viral persistence [12,16]. The fact that this sex-based disparity was most pronounced in younger individuals and markedly attenuated in those aged 50 years and older provides compelling, population-level support for a potential hormonal mechanism. The decline in estrogen levels during and after menopause could modulate immune responses, potentially reducing the sex-based advantage in viral clearance observed in younger, pre-menopausal women [16]. This life-course perspective suggests that the influence of sex on HCV outcomes is not static but may vary significantly with age, a critical consideration for both pathogenesis and programmatic planning.

The most programmatically significant finding was the substantially lower RNA positivity among young males (37.8%) than among young females (50.0%). This 32% relative difference suggests that young males represent a distinct subgroup where testing efficiency could be most dramatically improved. The 1.6-fold higher RNA positivity in adults aged 30 years and older further supports the consideration of age-informed testing approaches in resource-constrained settings. While our primary recommendation for a resource-constrained setting is a simple, age-informed algorithm prioritizing individuals ≥30 years (NNT=1.4) over those <30 years (NNT=2.2), our data reveal a further layer of stratification.

Within the young cohort itself, the NNT to find one viremic case is 2.7 for males versus 2.0 for females. This indicates that in settings with slightly more advanced diagnostic capacity or where maximizing efficiency is the absolute paramount concern, a more nuanced algorithm that de-prioritizes confirmatory testing for young seropositive males could be considered. The resources saved by this approach could be reallocated to further increase testing in high-yield groups. However, the wider confidence intervals around these estimates highlight the need for validation in larger cohorts before implementing such a sex-based triage within the youth demographic.

Our use of stringent laboratory safeguards-including elevated S/CO thresholds (≥5.0) and duplicate testing-strengthens confidence that the observed RNA negativity patterns reflect true biological variation rather than assay artifacts. The consistent genotype distribution (predominantly genotype 4) aligns with previous Ethiopian studies [7] and suggests that viral factors are less influential than host characteristics in determining RNA detectability in this population.

These findings lead to direct programmatic implications. In resource-constrained settings such as Ethiopia, testing efficiency is paramount for achieving HCV elimination goals [17]. Our data suggest that demographic-informed algorithms could optimize resource allocation. The low RNA positivity in young males (37.8%) indicates that this subgroup has a significantly lower pretest probability of viremia. Therefore, an approach that accepts a lower programmatic sensitivity in this demographically defined low-prevalence group is a pragmatic recalibration to accelerate the achievement of elimination goals, as saved resources can be reinvested into high-impact activities.

**Limitations:** several considerations should guide the interpretation of our findings. First, the retrospective cross-sectional design captures RNA status at a single time point and precludes causal inference about viral clearance dynamics. Second, clinical referral may have selected for symptomatic individuals, potentially inflating RNA positivity rates compared to community populations. Third, we lacked data on potential confounders, including duration of infection, HIV coinfection, comorbidities, and behavioral risk factors. Fourth, while age and sex were significant predictors, the moderate discriminatory performance (AUC=0.631) underscores that additional host, viral, and environmental factors influence RNA detectability, a finding consistent with HCV's complex natural history. Fifth, subgroup analyses, particularly for young males, had wider confidence intervals reflecting smaller sample sizes; these estimates should be considered preliminary until validated in larger cohorts. We presented multiple comparisons without formal statistical adjustment to provide complete analytical transparency.

**Generalizability:** the generalizability of our findings should be considered in context. Our results are most directly applicable to treatment-naïve, anti-HCV seropositive individuals undergoing confirmatory testing in Ethiopian urban clinical settings, particularly in referral laboratory networks similar to ours. The predominance of genotype 4 (49.6%) aligns with regional patterns in Ethiopia and neighboring countries [7], suggesting potential relevance to other East African settings with similar genotype distributions. However, caution is warranted when extrapolating to: (1) community-based populations not seeking healthcare, (2) regions with different HCV genotype distributions (e.g., West Africa with genotype 2 predominance), and (3) populations with different demographic structures or risk profiles. Our stringent laboratory criteria (elevated S/CO threshold, duplicate testing) enhance internal validity but may limit direct comparison with programs using standard manufacturer cutoffs. The efficiency gains suggested by our calculations should be validated in prospective implementation studies before widespread adoption. These findings provide a methodological template and evidence base for similar demographic-informed analyses in other resource-limited settings pursuing HCV elimination.

## Conclusion

This study confirms that both increasing age and female sex are independent demographic predictors of higher HCV RNA positivity among anti-HCV seropositive individuals in Ethiopia. The most programmatically significant finding is the substantially lower RNA detectability in young males (<30 years), which is 1.6-fold lower than in older adults. These findings imply that a one-size-fits-all testing algorithm is inefficient in resource-limited settings. A demographic-informed approach, which prioritizes confirmatory RNA testing for older individuals and females, could optimize resource allocation. The way forward requires prospective implementation research to validate the real-world efficiency and cost-effectiveness of such stratified testing strategies within national HCV elimination programs in Ethiopia and similar settings.

### What is known about this topic

- There is substantial variability in HCV RNA detectability among anti-HCV seropositive individuals, creating a diagnostic challenge in resource-limited settings;
- Younger age is generally associated with a higher rate of spontaneous clearance and lower RNA positivity, attributed to more robust immune responses;
- HCV genotype 4 is known to be the predominant genotype in Ethiopia.

### What this study adds

- This study provides population-specific evidence from a routine clinical setting in Ethiopia that female sex is an independent predictor of higher HCV RNA positivity, even after adjusting for age;
- It identifies young males (<30 years) as a distinct subgroup with exceptionally low RNA positivity (37.8%), highlighting a key opportunity for optimizing testing efficiency;
- It proposes a pragmatic, demographic-informed testing algorithm and quantifies its potential efficiency gains using the Number Needed to Test (NNT), offering a data-driven strategy for resource-limited elimination programs.
